# Effect of concentration, temperature, and time on the stability of hepatitis C virus nucleic acid in serum

**DOI:** 10.1128/spectrum.00769-24

**Published:** 2024-09-13

**Authors:** Ya-pei Wang, Shu-wen Yang, Fan Jia, Wei Wang, Xue Xu, Yi-xuan Wang, Jing-feng Bi, Bo-an Li

**Affiliations:** 1 Laboratory Department, Fifth Medical Center of PLA General Hospital, Beijing, China; 2 Grade 2022 Postgraduate Class, School of Pharmaceutical Sciences, Shandong University of Traditional Chinese Medicine, Jinan, China; 3 Phase I Clinical Trial Ward, Senior Department of Infectious Diseases Medicine, Fifth Medical Center of PLA General Hospital, National Clinical Research Center for Infectious Diseases, Beijing, China; 4 Grade 2021 Postgraduate Class, School of Pharmaceutical Sciences, Shandong University of Traditional Chinese Medicine, Jinan, China; University of Chicago, Chicago, Illinois, USA

**Keywords:** ribonuclease, hepatitis C virus nucleic acid, quality control

## Abstract

To explore the influence of storage temperature and time on the stability of different concentrations of hepatitis C virus nucleic acid (HCV RNA) samples and to provide data reference for laboratory quality control. Serum samples of 10 patients with HCV RNA detection quantitation of 10^6^–10^8^ IU/mL were collected. The samples of each patient were diluted into three concentrations: high, medium, and low. Then the samples of each concentration were divided into 21, which were divided into three groups according to the storage conditions of −20°C, 4°C, and 25°C, with seven samples in each group. The samples were selected from each group for quantitative detection of HCV RNA on day 0, day 1, day 3, day 5, day 7, day 14, and day 30. The results of each concentration and storage temperature sample remained stable within 5 days. Based on the mixed-effect linear model, the main effects of temperature, time, and concentration were statistically significant (*P* < 0.01). There was an interaction effect between concentration and time (*P* = 0.0448), and there was also an interaction effect between temperature and time (*P* < 0.01). There was no interaction effect between concentration and temperature (*P* = 0.11) or between concentration, temperature, and time (*P* = 0.90). The results of serum samples with different concentrations of the HCV RNA remained stable within 5 days. The lower the initial concentration of HCV RNA serum sample, the worse the stability; the higher the storage temperature, the worse the stability. If conditions permit, the laboratory should store such samples at −20°C.

**IMPORTANCE:**

Previously, there were few reports about the influence of different concentrations of sample nucleic acid on the stability of samples at various temperatures and times in various literatures. Therefore, it is necessary to analyze the influence of concentration factors on the stability of samples and test results at different storage times and temperatures. This study took the concentration of hepatitis C virus nucleic acid as the research object to further understand the stability of hepatitis C virus nucleic acid test samples under various storage conditions, to provide data reference for the treatment of hepatitis C virus nucleic acid and RNA test samples before clinical laboratory test, and provide guidance and help for the improvement of laboratory quality control.

## INTRODUCTION

Quantitative detection of hepatitis C virus RNA (HCV RNA) is the main index for clinical diagnosis of patients infected with hepatitis C virus and monitoring the therapeutic effect of patients with hepatitis C. Hepatitis C virus (HCV) belongs to single-stranded positive-strand RNA virus (1–5). HCV RNA structure is precarious, and the RNA in clinical test samples is vulnerable to degradation by endogenous ribonuclease RNase (6–9). Once the nucleic acid is degraded, the impact of the reduction of the extracted total nucleic acid will be multiplied by the nucleic acid amplification program in the real-time fluorescence quantitative detection process, resulting in the distortion of patient test results and affecting the quality of clinical diagnosis and treatment. Due to the consideration of test cost, staffing, and small sample size, most laboratories usually cannot do the on-demand test of samples when testing such samples. After receiving the samples, the laboratories need to store the samples in a certain temperature environment for some time. It is an important topic to understand the impact of sample storage conditions on the stability of samples.

At present, several studies have explored the influence of temperature and time on the stability of HCV RNA samples (10–23). However, we found in early clinical practice that the retest results of high-concentration samples after storage have a more stable trend than those of medium-concentration and low-concentration samples, especially with significant differences from those of low-concentration samples. The purpose of this study is to explore the influence of HCV RNA sample concentration on the retest results after preservation, and further clarify the comprehensive influence of concentration, time, and temperature on the retest stability. The project was approved by the ethics committee of the fifth medical center of the PLA General Hospital.

## MATERIALS AND METHODS

### Sample

#### Sample source

The samples were selected from patients with hepatitis C treated in the fifth medical center of the PLA General Hospital from February to March 2019, and 10 serum samples with hepatitis C virus nucleic acid detection quantitation of 10^6^–10^8^ IU/mL were collected.

Definition of concentration: since the upper limit of HCV RNA quantitative detection is generally 10^8^ IU/mL and the lower limit is 10^2^ IU/mL, 10^6^–10^8^ IU/mL is defined as high concentration, 10^4^–10^6^ IU/mL after 100 times dilution of 10^6^–10^8^ IU/mL sample is regarded as medium concentration, and 10^2^–10^4^ IU/mL after 100 times dilution of 10^4^–10^6^ IU/mL sample is regarded as low concentration.

### Sample inclusion criteria

Outpatients with a definite diagnosis of chronic hepatitis C and without treatment. Age > 18 years old, regardless of gender.

#### Sample exclusion criteria

1. Samples of patients with cross-infection of hepatitis B virus, HIV, respiratory virus, enterovirus, and other pathogens were excluded;
2. Samples of patients with diabetes, blood disease history and long-term medication history;
3. Samples of patients with autoimmune diseases and imaging diagnosis of liver cancer, cirrhosis or liver fibrosis;
4. The collected serum had obvious jaundice and fatty blood.

#### Sample grouping

**Fig 1:**
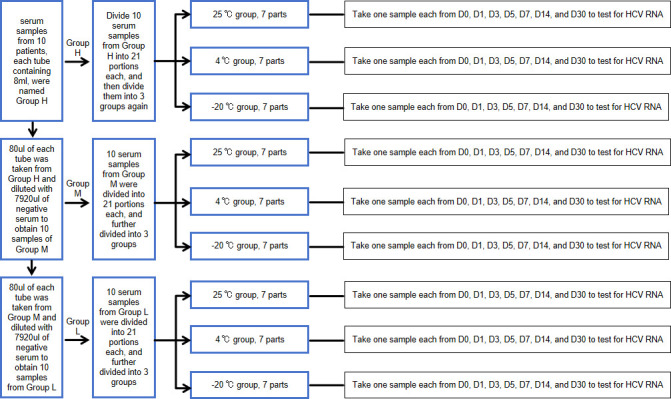
Sample sources. This figure mainly explains how to group the first 10 samples with a concentration of 10^6^–10^8^ IU/mL and when to detect them.

1. An amount of 7,920 μl of serum samples from 10 patients with nucleic acid detection quantitation of 10^6^–10^8^ IU/mL was, respectively, put into 10 mL sterile drying tubes, named high-concentration group (Group H), a total of 10 samples. See Fig. 1 for grouping details.
2. Take 80 μl of the remaining serum samples from 10 patients whose nucleic acid detection quantitation is 10^6^–10^8^ IU/mL, respectively, and put them into 10 mL sterile drying tubes. Then add 7,920 μl of healthy human serum into each tube, and fully mix to obtain a medium-concentration (10^4^–10^6^ IU/mL) sample, a total of 10 samples.
3. An amount of 7,920 μl of 10 samples with medium concentration (10^4^–10^6^ IU/mL) were, respectively, put into 10 mL sterile drying tubes, which were named as medium-concentration group (Group M), a total of 10 samples.
4. Take the remaining 80 μl from 10 samples of medium concentration (10^4^–10^6^ IU/mL) and put them into 10 mL sterile drying tubes, respectively. Then add 7,920 μl of healthy human serum into each tube and mix well to obtain a low concentration (10^2^–10^4^ IU/mL) group (Group L), a total of 10 samples, 8 mL each.
5. Each sample in Group H was further divided into 21 pieces and put into the EP tube of one-time inactivated RNase (about 370 µL per tube). They were randomly divided into three groups, with seven pieces in each group. They were stored at 25°C, 4°C, and −20°C, respectively. The HCV RNA concentration was detected immediately after grouping (before preservation), 24 hours (1 day), 72 hours (3 days), 5 days, 7 days, 14 days, and 30 days.
6. Group M and Group L were grouped and detected in the same way as Group H.

### HCV RNA extraction

In this study, the magnetic bead method was used to extract nucleic acid, and the HCV RNA nucleic acid extraction kit and HCV RNA amplification kit of Hunan Shengxiang Biotechnology Co., Ltd., the Natch S automatic nucleic acid extraction instrument and equipment computer were used. The protein in the sample is cleaved by the nucleic acid-releasing agent, and the hepatitis C virus nucleic acid RNA is released at the same time. The surface of the magnetic beads is coated with functional groups that can adsorb the function of hepatitis C virus nucleic acid RNA. The hepatitis C virus nucleic acid RNA can be adsorbed on the surface of the magnetic beads. After repeated washing, impurities and large proteins are removed, and finally high-purity hepatitis C virus nucleic acid is obtained. After the extracted nucleic acid was mixed with the reaction solution, the PCR plate was removed, covered with the sealing film, centrifuged, and transferred to the Roche light cycle Z480 PCR amplification instrument, and the amplification program was selected for nucleic acid amplification.

### HCV RNA amplification

In the setup panel of the instrument operation software interface of Roche light cycle z480 device computer, set the HCV nucleic acid, the PCR template. The temperature control procedure of PCR was as follows: (1) pre-denaturation and enzyme activation were 95°C, lasting for 1 minute, and the number of cycles was 1; (2) reverse transcription was performed at 60°C for 30 minutes, and the number of cycles was 1; (3) The cDNA was pre-denatured at 95°C for 1 minute, and the number of cycles was 1; (4) denaturation at 95°C for 15 seconds; annealing extension and fluorescence detection at 60°C, 30 seconds; the number of cycles in this step is 45; and (5) the instrument is cooled at 25°C for 10 seconds, and the number of cycles is 1. Using a pair of specific primers and a specific fluorescent probe designed for the conserved region of hepatitis C virus RNA, combined with PCR solution, the real-time fluorescent quantitative PCR detection technology was applied to the real-time fluorescent quantitative PCR instrument to realize the quantitative detection of hepatitis C virus RNA through the change in fluorescent signal.

### Judgment of effective results

1. The amplification curves of all samples were by S-shaped curve;
2. The internal standard curve should be in a reasonable Cycle interval;
3. The amplification curves of standard samples were equally spaced and consistent with the sample curves;
4. The amplification efficiency of standard reference amplification results should be within the range of 2.0 ± 0.1, the error rate should be less than 0.01, and the linear slope should be within the range of −3.3 ± 0.1; The detection results of positive control samples are within ±1 SD of the target value, and the negative control samples have no amplification signal.

For the samples whose individual expected test results are too different from the actual test results and the samples whose results cannot be judged in the experiment, it was necessary to re-check and confirm on the third-party instruments and equipment (such as Roche Cobas cap), and finally determine the sample test results based on the re-inspection results, test results and expected results.

### Statistical methods

In this study, samples with different concentrations, temperatures, and times were tested from the same source, belonging to the block design. Before statistical analysis, the quantitative results of HCV RNA were first transformed into a logarithm. When the data conformed to the normality and homogeneity of variance, it was expressed as mean ± standard deviation. The overall comparison between groups was performed by block design analysis of variance, and the pairwise comparison was performed by SNK-q test; when the data do not conform to the normality or variance homogeneity, it is expressed by the median (quartile). The overall comparison between groups is performed by the Friedman rank sum test, and the pairwise comparison is performed by the Wilcoxon signed rank test.

The mixed-effect linear model was used to analyze the results of HCV RNA samples at different concentrations, temperatures, and times. Using SAS9.4 statistical analysis software, all statistical analysis processes were taken bilateral test, and *P* < 0.05 was judged as statistically significant.

## RESULTS

### Baseline at D0

The sample concentration baseline information (Table 1).

**TABLE 1:** Baseline at D0

Concentration	Temperature		
	25°C	4°C	−20°C
Group H	6.91 (6.57, 7.51)	6.90 (6.60, 7.53)	6.89 (6.58, 7.53)
Group M	4.85 (4.62, 5.47)	4.84 (4.63, 5.49)	4.83 (4.61, 5.48)
Group L	2.79 (2.51, 3.22)	2.79 (2.53, 3.30)	2.78 (2.52, 3.32)

### Effect of different concentrations on the stability of HCV RNA

The results showed that at 25°C, from D7, there was a significant difference in the concentration of HCV RNA among the initial high-, medium-, and low-concentration groups; at 4°C, from D14, there was a significant difference in the concentration of HCV RNA among the initial high-, medium,- and low-concentration groups; at −20°C, there was a significant statistical difference in the concentration of HCV RNA among the initial high-, medium-, and low-concentration groups of D7 and D14. The above suggests that the reduction of HCV RNA concentration in the initial low-concentration group is significantly greater than that in the high-concentration group and low-concentration group (see Table 2), indicating that the initial concentration of HCV RNA has a great impact on the concentration change after long-term storage, and the lower the concentration, the greater the impact.

**TABLE 2:** Comparison of results of samples at different temperatures under different concentrations

Temperature	Time	Concentration			Statistic	*P* value
		Group H	Group M	Group L		
25°C	D1	−0.01 (−0.04, 0.06)	−0.00 (−0.04, 0.02)	0.02 (0.00, 0.05)	2.40	0.30
	D3	0.01 (−0.04, 0.05)	0.03 (−0.02, 0.05)	0.04 (0.03, 0.08)	4.20	0.12
	D5	0.04 (−0.08, 0.09)	0.02 (−0.05, 0.06)	0.06 (0.02, 0.09)	3.20	0.20
	D7	0.08 (0.04, 0.11)	0.02 (−0.01, 0.06)^*b*^	0.12 (0.05, 0.14)	7.20	0.03
	D14	0.14 (0.07, 0.16)	0.07 (0.00, 0.11)	0.19 (0.15, 0.20)	6.20	0.05
	D30	0.13 (0.09, 0.18)^*a*^	0.13 (0.06, 0.15)^*b*^	0.41 (0.23, 0.42)	13.40	0.00
4°C	D1	0.02 (−0.01, 0.04)	−0.01 (−0.02, 0.02)	0.03 (0.01, 0.05)	4.20	0.12
	D3	0.01 (−0.03, 0.02)	0.00 (−0.04, 0.02)	0.01 (−0.03, 0.05)	1.80	0.41
	D5	0.03 (−0.00, 0.04)	0.00 (−0.01, 0.03)	0.03 (−0.01, 0.03)	1.40	0.50
	D7	0.02 (−0.00, 0.06)	−0.01 (−0.03, 0.03)	0.0 4 (0.02, 0.08)	5.60	0.06
	D14	0.04 (0.01, 0.06)^*a*^	0.04 (0.02, 0.08)^*b*^	0.09 (0.07, 0.12)	12.20	0.00
	D30	0.05 (0.02, 0.07)^*a*^	0.05 (0.02, 0.06)^*b*^	0.13 (0.10, 0.20)	9.80	0.01
−20°C	D1	−0.01 (−0.01, 0.00)	−0.02 (−0.04, 0.02)	0.00 (−0.00, 0.02)	3.80	0.15
	D3	0.01 (−0.01, 0.03)	0.01 (−0.01, 0.02)	0.02 (0.01, 0.04)	2.60	0.27
	D5	0.01 (0.01, 0.03)	0.02 (−0.01, 0.03)	0.02 (0.01, 0.05)	1.40	0.50
	D7	0.01 (−0.01, 0.04)	0.00 (−0.02, 0.01)^*b*^	0.03 (0.02, 0.03)	9.80	0.01
	D14	0.00 (−0.01, 0.02)^*a*^	0.01 (0.01, 0.05)	0.04 (0.03, 0.06)	6.20	0.04
	D30	0.01 (0.00, 0.05)	0.03 (0.01, 0.04)	0.04 (0.02, 0.05)	1.80	0.40

^*a*^ Means that there is a significant difference between Group H and Group L (*P* ≤ 0.05).

^*b*^ Means that there is a significant difference between Group M and Group L (*P* ≤ 0.05).

### Effect of storage temperature on the stability of HCV RNA

The results showed that the high-concentration group showed statistical differences between the three groups of different temperatures from D7 (D7, *P* = 0.01; D14, *P* = 0.01; D30, *P* < 0.01), mainly reflected in the differences between the 25°C group and the 4°C group, as well as the differences between the 25°C group and the −20°C group. Combined with the specific values of concentration changes, the changing trend of the 25°C group >4°C group >−20°C group could be found at D5; in the medium-concentration group, there was a statistical difference between the three groups of different temperatures at D14 (D14, *P* = 0.02; D30, *P* < 0.01), which was mainly reflected in the differences between the 25°C group and the 4°C group, as well as the differences between the 25°C group and the −20°C group. Combined with the specific value of concentration change, the changing trend of the 25°C group >4°C group >−20°C group could be found at D14; the low-concentration group began to show statistical differences among the three groups at D5 (D5, *P* = 0.04; D7, *P* = 0.01; D14, *P* < 0.01; D30, *P* < 0.01). Among them, D5-D14 mainly reflected the differences between the 25°C group and the 4°C group and the −20°C group, and D30 also reflected the differences between the 4°C group and the −20°C group. Combined with the specific values of concentration changes, the changing trend of the 25°C group >4°C group >−20°C group could be found at D5 (see Table 3). These results suggest that the change trend of HCV RNA concentration stored at 25°C was the largest, while the change trend of HCV RNA concentration stored at −20°C was the smallest, that is, with the decrease in storage temperature, the concentration of HCV RNA had a more stable trend.

**TABLE 3:** Concentration changes of each group of samples under different temperatures

Concentration	Time	Temperature			Statistic	*P* value
		25°C	4°C	−20°C		
Group H	D1	−0.01 (−0.04, 0.06)	0.02 (−0.01, 0.04)	−0.01 (−0.01, 0.00)	2.60	0.27
	D3	0.01 (−0.04, 0.05)	0.01 (−0.03, 0.02)	0.01 (−0.01, 0.03)	1.40	0.50
	D5	0.04 (−0.08, 0.09)	0.03 (−0.00, 0.04)	0.01 (0.01, 0.03)	0.20	0.90
	D7	0.08 (0.04, 0.11)^*a*^^*b*^	0.02 (−0.00, 0.06)	0.01 (−0.01, 0.04)	9.80	0.01
	D14	0.14 (0.07, 0.16)^*a*^	0.04 (0.01, 0.06)	0.00 (−0.01, 0.02)	8.60	0.01
	D30	0.13 (0.09, 0.18)^*a*^^*b*^	0.05 (0.02, 0.07)	0.01 (0.00, 0.05)	14.60	＜0.01
Group M	D1	−0.00 (−0.04, 0.02)	−0.01 (−0.02, 0.02)	−0.02 (−0.04, 0.02)	1.40	0.50
	D3	0.03 (−0.02, 0.05)	0.00 (−0.04, 0.02)	0.01 (−0.01, 0.02)	1.40	0.50
	D5	0.02 (−0.05, 0.06)	0.00 (−0.01, 0.03)	0.02 (−0.01, 0.03)	0.20	0.90
	D7	0.02 (−0.01, 0.06)	−0.01 (−0.03, 0.03)	0.00 (−0.02, 0.01)	5.60	0.06
	D14	0.07 (0.00, 0.11)^*b*^	0.04 (0.02, 0.08)	0.01 (0.01, 0.05)	7.80	0.02
	D30	0.13 (0.06, 0.15)^*a*^^*b*^	0.05 (0.02, 0.06)	0.03 (0.01, 0.04)	15.20	0.00
Group L	D1	0.02 (0.00, 0.05)	0.03 (0.01, 0.05)	0.00 (−0.00, 0.02)	3.80	0.15
	D3	0.05 ± 0.07	0.00 ± 0.04	0.02 ± 0.02	1.58	0.19
	D5	0.06 (0.02, 0.09)^*b*^	0.03 (−0.01, 0.03)	0.02 (0.01, 0.05)	6.20	0.04
	D7	0.12 (0.05, 0.14)^*b*^	0.04 (0.02, 0.08)	0.03 (0.02, 0.03)	9.80	0.01
	D14	0.16 ± 0.10^*a*^^*b*^	0.10 ± 0.03	0.04 ± 0.03	4.77	0.00
	D30	0.41 (0.23,0.42)^*a*^^*b*^	0.13 (0.10,0.20)^*c*^	0.04 (0.02, 0.05)	20.00	＜.0001

^*a*^ Means that there is a significant difference between 25°C group and 4°C group (*P* < 0.05).

^*b*^ Means that there is a significant difference between 25°C group and −20°C group (*P* < 0.05).

^*c*^ Means that there is a significant difference between 4°C group and −20°C group (*P* < 0.05).

### Effect of storage time on the stability of HCV RNA

The results showed that compared with D0, in the 25°C group, the high concentration had a significant statistical difference from D7, the medium concentration had a significant statistical difference from D14, and the low concentration had a significant statistical difference from D5, suggesting that it is appropriate to store HCV RNA at 25°C for no more than 3 days without considering the effect of concentration; Compared with D0, in the 4°C group, the high concentration of D30 had significant statistical difference, the medium concentration of D30 had significant statistical difference, and the low concentration had significant statistical difference from D14, suggesting that it is appropriate to store HCV RNA at 4°C for no more than 7 days without considering the effect of concentration; Compared with D0, there was no significant statistical difference in the −20°C group at high, medium, or low concentrations, suggesting that HCV RNA can be stored at −20°C for at least 30 days (see Table 4). These results showed that the concentration of HCV RNA decreased with the extension of storage time, and the longer the storage time, the more obvious the decrease trend of HCV RNA.

**TABLE 4:** Comparison of sample results at different times under different concentrations and temperatures

Concentration	Temperature	Time					
		D1	D3	D5	D7	D14	D30
Group H	25°C	−0.01 (−0.04, 0.06)	0.01 (−0.04, 0.05)	0.04 (−0.08, 0.09)	0.08 (0.04, 0.11)^*a*^	0.14 (0.07, 0.16)^*a*^	0.13 (0.09, 0.18)^*a*^
	4°C	0.02 (−0.01, 0.04)	0.01 (−0.03, 0.02)	0.03 (−0.00, 0.04)	0.02 (−0.00, 0.06)	0.04 (0.01, 0.06)	0.05 (0.02, 0.07)^*a*^
	−20°C	−0.01 (−0.01, 0.00)	0.01 (−0.01, 0.03)	0.01 (0.01, 0.03)	0.01 (−0.01, 0.04)	0.00 (−0.01, 0.02)	0.01 (0.00, 0.05)
Group M	25°C	−0.00 (−0.04, 0.02)	0.03 (−0.02, 0.05)	0.02 (−0.05, 0.06)	0.02 (−0.01, 0.06)	0.07 (0.00, 0.11)^*a*^	0.13 (0.06, 0.15)^*a*^
	4°C	−0.01 (−0.02, 0.02)	0.00 (−0.04, 0.02)	0.00 (−0.01, 0.03)	−0.01 (−0.03, 0.03)	0.04 (0.02, 0.08)	0.05 (0.02,0.06)^*a*^
	−20°C	−0.02 (−0.04, 0.02)	0.01 (−0.01, 0.02)	0.02 (−0.01, 0.03)	0.00 (−0.02, 0.01)	0.01 (0.01, 0.05)	0.03 (0.01, 0.04)
Group L	25°C	0.02 (0.00, 0.05)	0.04 (0.03, 0.08)	0.06 (0.02, 0.09)^*a*^	0.12 (0.05, 0.14)^*a*^	0.19 (0.15, 0.20)^*a*^	0.41 (0.23, 0.42)^*a*^
	4°C	0.03 (0.01, 0.05)	0.01 (−0.03, 0.05)	0.03 (−0.01, 0.03)	0.04 (0.02, 0.08)	0.09 (0.07,0.12)^*a*^	0.13 (0.10, 0.20)^*a*^
	−20°C	0.00 (−0.00, 0.02)	0.02 (0.01, 0.04)	0.02 (0.01, 0.05)	0.03 (0.02, 0.03)	0.04 (0.03, 0.06)	0.04 (0.02, 0.05)

^*a*^ Means that there is a statistical difference between the time and day 0 (*P* ≤ 0.05).

### Multivariate analysis of temperature, time, and concentration

Multivariate analysis of temperature, time, and concentration based on a mixed-effect linear model showed that the main effects of temperature, time, and concentration were statistically significant (*P* < 0.01). There was an interaction effect between concentration and time (*P* = 0.0448), and there was also an interaction effect between temperature and time (*P* < 0.01). There was no interaction effect between concentration and temperature (*P* = 0.11), and there was no interaction effect between concentration, temperature, and time (*P* = 0.90) (see Table 5).

**TABLE 5:** Overall analysis of all variables

Effect	F value	*P* value
Concentration	113,348	＜0.01
Temperature	16.23	＜0.01
Time	16.67	＜0.01
Concentration*temperature	1.91	0.11
Concentration*time	1.80	0.04
Temperature*time	4.19	＜0.01
Concentration*temperature*time	0.65	0.90

## DISCUSSION

In this study, the stability of HCV RNA samples was studied by three factors: temperature, time, and concentration. In the process of the study, we diluted the collected samples to obtain three different concentration groups: high, medium, and low. Each concentration group was divided into 21 samples again. At the same time, each group of samples was divided into three groups (seven in each group) and stored at 25°C, 4°C, and −20°C, respectively. According to the storage time of 0, 1, 3, 5, 7, 14, and 30 days, one sample was taken from the different storage temperature groups of each concentration group for HCV RNA nucleic acid detection. In the detection process, nucleic acid extraction by magnetic beads and PCR amplification technology was used to quantify HCV RNA, take the log value of quantitative results, and summarize them for analysis. The results showed that the quantitative results of HCV RNA began to show statistical differences among the groups after the samples were stored for 5 days. With the extension of time, the statistical differences between the control groups gradually increased, and the differences were concentrated between the low-concentration samples and the medium- and high-concentration samples. Especially, the results of the low-concentration samples stored at 25°C on the 30th day exceeded the range of ±0.4 of the logarithm of the results set by the quality control on the 0th day. On the one hand, the stability of the low-concentration samples was worse than that of the medium- and high-concentration samples, and on the other hand, the stability of the samples gradually deteriorated after 5 days of storage, especially the stability of the samples stored at 25°C was significantly worse than that of 4°C and −20°C. In addition, the mixed effect linear model analysis of variables showed that the lower the initial concentration of HCV RNA serum sample, the worse the stability. The higher the storage temperature, the worse the stability, and the longer the storage time, the worse the stability.

All serum samples in this study could maintain good stability within 5 days of storage. Although the results of each group began to differ in varying degrees on the fifth day, according to the relevant requirements of CNAS-CL02-A001:2021 *Application Instructions of Medical Laboratory Quality and Ability Accreditation Criteria in the Field of Molecular Diagnosis* (24), the differences between groups still did not exceed the range of quality control (target value ±0.4) on the 14th day, and the differences between groups on the 14th day and 30th day gradually increased, indicating that the influence of various factors on the stability of HCV RNA began to appear. In particular, the results of low-concentration samples are most affected by time and temperature in this study, which also reminds our laboratory operators to pay special attention to the treatment of low-concentration samples. The storage temperature and storage time designed in this study are in line with the actual detection conditions of most laboratories at present, and the detection methods, instruments equipment, and detection reagents currently used by various laboratories for HCV RNA quantification are also similar to this study. Therefore, the results of this study can truly reflect the sample stability problems that various laboratories may face when processing such samples, which provides a reference for the evaluation of sample stability and detection quality in the current laboratory.

Relevant studies and reports have been conducted at home and abroad on the impact of RNA sample processing under different conditions on sample stability and detection results. According to the research conclusions reported in the literature (25–34), most researchers have reached a consensus in some aspects of this field, such as the use of fluorescent quantitative PCR for clinical diagnosis (35), the need for frozen RNA samples (36–38), etc. Through reading a large number of reports in the literature, this study found that for the study of the storage time on the stability of RNA, Marta Jos é et al. (13) reported that the nucleic acid concentration of HCV RNA with a concentration of 100 IU/mL of hepatitis C virus in serum remained stable at −20°C for 5 years. Han Yanan (10) and Yin Yundi (22) reported that there were differences in the samples stored at 4°C from the 7th day. Sener K (13) reported that HCV RNA in blood samples stored at 4°C could be kept stable for 5 weeks. Therefore, the conclusions of previous studies were different. The results of the samples stored at 4°C in this study began to show statistical differences among the groups on the 14th day, and with the extension of time, the frequency of differences increased, and the stability of the samples tended to deteriorate gradually, so the best detection period at 4°C was within 14 days. As for the study on the stability of samples under storage temperature, Baleriola c (18) and others tested HCV RNA samples stored at −20°C and −70°C for 9 years. The results showed that the loss of nucleic acid was within 10 IU/mL, which reflected that low temperature storage was very important for the stability of samples, but it was impossible to store samples for a long time in the process of laboratory work practice, and repeated freezing and thawing of samples would also increase the risk of RNA damage. For the analysis of the test results of high-, medium-, and low-concentration samples after storage, it can be found that the difference of low-concentration samples is significantly higher than that of high-concentration samples, and the stability of samples stored at 25°C is also worse than that at 4°C and −20°C. Lei Xiuxia (39) believed that when the content of HCV RNA in blood samples is high (>10^5^ IU/mL), RNA is more stable, which is consistent with the conclusion of the stability of high-concentration samples in this study. Zhuang Yanglin et al. found that HCV RNA samples degraded after 48 hours of storage at room temperature and 25°C, and HCV RNA could not be detected after 4 weeks of storage (11). Liu changli et al. found that the virus content decreased to 72.5% of the original titer after 48 hours of storage at 25°C. The above conclusion showed that the stability of HCV RNA samples at room temperature was poor, which was different from the results of this study.

Compared with previous studies, the laboratory software and hardware environment have developed rapidly in recent years, especially with the wide application of magnetic bead nucleic acid extraction technology and automatic nucleic acid extraction equipment, the standardization of laboratory standardized operation process and the improvement of quality management awareness of laboratory personnel, and the inspection quality monitoring has run through the whole process before, during, and after the inspection. Therefore, the external influencing factors of HCV RNA samples have been continuously reduced. It is necessary to re-explore the storage conditions of HCV RNA samples in the laboratory. In addition, this study included high concentration, medium concentration, and low concentration in the research content for the first time, which has not been reported in-depth in previous studies, especially taking temperature, time, and concentration as the research objects at the same time, which is the first time in the study of HCV RNA sample stability.

The limitations of this study are as follows: (i). The research object of this study is only for serum samples, and the stability of HCV RNA in plasma, whole blood, tissue, and other body fluid samples is not studied. The research conclusion cannot be compared with a similar research conclusion with plasma, whole blood, and tissue samples as the research object. (ii) This study did not involve factors such as high jaundice and hyperlipidemia in the sample serum, nor did it include disease factors such as cross-infection of multiple pathogens, drug-induced effects, other chronic diseases, blood diseases, and autoimmunity in this study. On the one hand, it was difficult to collect such samples, on the other hand, it took into account the factors such as research cycle and research cost.

### Conclusion

Our study found that the lower the initial concentration of HCV RNA serum samples, the worse the stability. The higher the storage temperature, the worse the stability, and the longer the storage time, the worse the stability. When the initial concentration of HCV RNA in the sample is low, the stability of HCV RNA is poor, and short-term storage at room temperature will affect the stability of viral nucleic acid. Therefore, great attention should be paid to the storage of low-concentration samples. The serum samples for HCV RNA detection are most affected by the storage temperature. If they cannot be detected in time, they should be centrifuged and transferred to a 4°C refrigerator for short-term storage, preferably no more than 5 days. For the samples that need to be stored for a long time, the laboratory with permission will separate the serum from these samples and store them at −20°C to ensure the stability of the nucleic acid of the samples.
